# Application of the National Institute for Health and Care Excellence Evidence Standards Framework for Digital Health Technologies in Assessing Mobile-Delivered Technologies for the Self-Management of Type 2 Diabetes Mellitus: Scoping Review

**DOI:** 10.2196/23687

**Published:** 2021-02-16

**Authors:** Jessica R Forsyth, Hannah Chase, Nia W Roberts, Laura C Armitage, Andrew J Farmer

**Affiliations:** 1 Medical Sciences Division University of Oxford Oxford United Kingdom; 2 Bodleian Health Care Libraries University of Oxford Oxford United Kingdom; 3 Nuffield Department of Primary Care Health Sciences University of Oxford Oxford United Kingdom

**Keywords:** type 2 diabetes, health technology, self-management, mobile health, mobile applications, guidelines

## Abstract

**Background:**

There is a growing role of digital health technologies (DHTs) in the management of chronic health conditions, specifically type 2 diabetes. It is increasingly important that health technologies meet the evidence standards for health care settings. In 2019, the National Institute for Health and Care Excellence (NICE) published the *NICE Evidence Standards Framework for DHTs*. This provides guidance for evaluating the effectiveness and economic value of DHTs in health care settings in the United Kingdom.

**Objective:**

The aim of this study is to assess whether scientific articles on DHTs for the self-management of type 2 diabetes mellitus report the evidence suggested for implementation in clinical practice, as described in the *NICE Evidence Standards Framework for DHTs*.

**Methods:**

We performed a scoping review of published articles and searched 5 databases to identify systematic reviews and primary studies of mobile device–delivered DHTs that provide self-management support for adults with type 2 diabetes mellitus. The evidence reported within articles was assessed against standards described in the NICE framework.

**Results:**

The database search yielded 715 systematic reviews, of which, 45 were relevant and together included 59 eligible primary studies. Within these, there were 39 unique technologies. Using the NICE framework, 13 technologies met *best practice* standards, 3 met *minimum* standards only, and 23 technologies did not meet *minimum* standards.

**Conclusions:**

On the assessment of peer-reviewed publications, over half of the identified DHTs did not appear to meet the minimum evidence standards recommended by the NICE framework. The most common reasons for studies of DHTs not meeting these evidence standards included the absence of a comparator group, no previous justification of sample size, no measurable improvement in condition-related outcomes, and a lack of statistical data analysis. This report provides information that will enable researchers and digital health developers to address these limitations when designing, delivering, and reporting digital health technology research in the future.

## Introduction

### Background

Digital technologies are now integral to the delivery of health care and feature in policies for the future of national [1] and global [2] health care systems. The World Health Organization (WHO) defines a health technology as “the application of organized knowledge and skills in the form of devices, medicines, vaccines, procedures and systems, developed to solve a health problem and improve quality of lives” [3]. Typically, digital health technologies (DHTs) include apps, software, and web-based platforms intended to benefit people or the wider health care system [4]. DHTs are increasingly supporting or being used as an adjunct to face-to-face clinical care by facilitating remote health care.

Many DHTs are intended to support chronic disease management, where self-management and preventative medicine are key components of effective care. Approximately 500 million people use mobile device apps to manage their health [5], and diabetes is the condition most commonly targeted by commercial apps [6]. With an increasing global prevalence of type 2 diabetes, mobile device apps offer a potential means of supporting diabetes care, particularly in the context of increasing demands against limited resources. It is imperative that the quality, safety, and effectiveness of such mobile device apps are assessed before deployment in clinical practice. In 2019, the WHO cautioned that amid increasing interest, digital health has been characterized by interventions being implemented without careful examination of the evidence base on their benefit and harms [7]. In the same year, the National Institute for Health and Care Excellence (NICE) published the Evidence Standards Framework for DHTs to guide clinicians, researchers, and policy makers in assessing whether the published literature evaluating these technologies provides the required level of evidence for their intervention to be considered for use in the UK health care setting [4].

There are several existing guidelines on evaluating the use of DHTs, including guidelines by policy makers such as the WHO, the United States’ Federal Drug Association, and National Health Service England [8-11] as well as frameworks developed by independent research groups [12,13]. However, the NICE framework is unique in explicitly suggesting a quality standard in relation to a technology’s functionality. Although the NICE framework was developed for DHTs used in a UK health care setting, the framework has the advantage of being research oriented rather than reliant on nation-specific commercial standards. This provides an opportunity for applying the framework to broader settings. First, the research-based focus may allow the framework to be used to evaluate the effectiveness of both consumer-driven and clinician-prescribed DHTs. Second, the framework may also be adapted to other health care systems by adjusting the requirement for development and testing in the United Kingdom to that of the DHT’s *host country*. Therefore, the NICE Evidence Framework may be used to guide assessment of and make comparisons between scientific literature regarding a variety of DHTs developed and applied internationally.

The NICE framework classifies apps by function and stratifies them into tiers (tiers 1, 2, 3a, or 3b). The tier framework corresponds with the evidence level required to support use of the technology; requirements are cumulative, becoming increasingly rigorous from tier 1 to 3 and divided into *best practice* and *minimum* standards. Stakeholders are encouraged to assess the evidence against these standards, which include, for example, whether the study measures important outcomes for users, whether the intervention works independently of health care professionals’ input, and the extent to which the intervention guides diagnosis, management, and treatment of a disease.

To date, there has been no review exploring whether peer-reviewed scientific literature regarding DHTs meets these evidence requirements. We investigated this in the context of DHTs designed to support the self-management of type 2 diabetes, as it is the most common chronic condition targeted by self-management DHTs [6].

### Objectives

The objectives of this review are (1) to systematically identify peer-reviewed publications on mobile device DHTs intended to support or encourage the self-management of type 2 diabetes mellitus (T2DM), (2) to use the NICE Evidence Standards Framework to allocate each DHT to the appropriate intervention tier based on their described technology and function, and (3) to examine the extent to which the evidence reported for the identified DHTs meets the NICE framework level of evidence required according to its tier.

## Methods

### Review Design

We performed a scoping review [14] to understand the literature to date and explore the application of research methodology in relation to the NICE evidence standards. The review is reported according to the Preferred Reporting Items for Systematic Reviews and Meta-Analyses (PRISMA) statement [15]. 

### Data Sources

A total of 5 databases (MEDLINE, Embase, PsycINFO, CINAHL, and Cochrane Database of Systematic Reviews) were searched for systematic reviews published between January 2000 and August 2019 that evaluated mobile device DHT interventions for T2DM. Our database choice and search strategy were developed through consultation with a medical information specialist to identify the most relevant sources for peer-reviewed medical and clinical research studies. An example search strategy is provided in Multimedia Appendix 1.

### Screening for Systematic Reviews

Two reviewers (JF and LA) independently screened all citations for systematic reviews by title and abstract and excluded those that clearly did not meet the eligibility criteria. Decisions were then unblinded, and any conflicting decisions were arbitrated by a third reviewer (AF). Full-text articles for all included citations were then screened against the inclusion criteria by 2 reviewers (JF and LA). 

Reviews were eligible if they included primary studies evaluating mobile apps designed to support adults with the self-management of diabetes mellitus. Reviews were excluded if they included studies in which the study population included people with type 1 diabetes, an undifferentiated mix of people with type 1 diabetes or type 2 diabetes, gestational diabetes, childhood diabetes or prediabetes, or focused on diagnosing diabetes (due to our focus on assessing DHTs designed to support self-management). Reviews that focused exclusively on telemedicine or telehealth interventions were also excluded, owing to our focus on technologies that support self-management and therefore require some degree of functionality independent of a clinician.

### Screening for Primary Studies and Technologies

Relevant primary studies were then identified from eligible systematic reviews. The eligible reviews were equally divided between the 4 reviewers (JF, LA, HC, and AF) who then screened the title and abstract of each primary study included in each review. When a primary study was excluded, the study was double screened by a second reviewer, and in the instance of any conflict, a third reviewer arbitrated (LA or AF). Primary studies included at this stage were then divided between the 4 reviewers who reviewed the full text of each study for eligibility. Furthermore, when a study was excluded, the study was double screened by a second reviewer, and any conflict was arbitrated by a third reviewer (LA or AF).

Primary studies were eligible for inclusion if they met the following inclusion criteria:

1. Population: adults with a diagnosis of T2DM.
2. Intervention: a mobile device–delivered DHT designed to support the self-management of T2DM, which provides support independent of a clinician.

### Data Extraction

Data were extracted from the included primary studies by 4 reviewers (JF, LA, HC, and AF). We designed a custom data extraction form using the *evidence for effectiveness tables* from the NICE framework [4] and additional guidance in the framework; an explanation of this approach can be found in Multimedia Appendix 2.

We extracted the following items from primary studies: (1) DHT investigated, (2) year of study, (3) study nation, (4) study design, (5) study setting, (6) outcomes of interest, (7) study duration and follow-up period, (8) sample size, (9) recruitment setting, (10) comparator group, (11) improvement in outcome with intervention, (12) justification of sample size, (13) statistical methods, and (14) follow-up rate. For tier 3a studies, we also extracted the following item: (15) description of and reference to a behavior change technique. Where more than one article that investigated the same DHT intervention was identified, data were extracted separately for each article.

### Assigning Technologies and Intervention Tier

Descriptions of each technology were extracted from the primary studies, and we assigned each app a tier according to the NICE framework, as described in Multimedia Appendix 2. Where an app had more than one function, the function with the highest applicable tier was considered when assigning an overall tier. Tier 3b was considered as a higher tier to 3a owing to its more rigorous evidence requirements, as detailed in Multimedia Appendix 2.

### Assessment of Evidence According to Tier

We used the NICE framework to evaluate each DHT against evidence levels, referring to evidence in the primary studies for each DHT, as described in Multimedia Appendix 2. We assessed each technology against its highest relevant tier to determine whether the DHT met the framework’s *minimum* and *best practice* evidence requirements. Where a technology was reported in more than one primary study, we analyzed each primary study separately against the framework and selected the strongest supporting evidence for the technology reported across the primary studies.

We also compared the NICE evidence standards outcome for a DHT against the income status of the study nation (as defined by the World Bank [16]). This was done to explore whether the NICE framework could be applied to DHTs designed for a different health care structure and system outside of the United Kingdom; a need for more empirical approaches to assess DHTs in low- and middle-income countries has been highlighted in recent literature [17,18].

Tier 3a guidance requires evidence of a referenced behavioral change technique (BCT) in the development or use of a technology that encourages behavioral change. For the purposes of this review and evidence assessment, we took a pragmatic decision to exclude this requirement in our overall decision on whether a tier 3a technology met the evidence requirements, accounting for the fact that our search methods may not have identified all relevant development studies reporting on a technology’s design.

In addition, the framework defines *data quality* as the presence of “statistical considerations such as sample size and statistical testing.” A pragmatic decision was made that statistical testing of some degree was needed as the *minimum* evidence requirement for all studies. However, the framework accommodates observational and quasi-experimental study designs, where it is impractical to statistically justify the sample size. Therefore, when making an assessment of evidence for studies of these designs, a statistical justification of sample size was not needed to meet *minimum* standards (but was required for experimental studies or randomized controlled trials [RCTs]).

## Results

### Screening for Systematic Reviews

**Figure 1:**
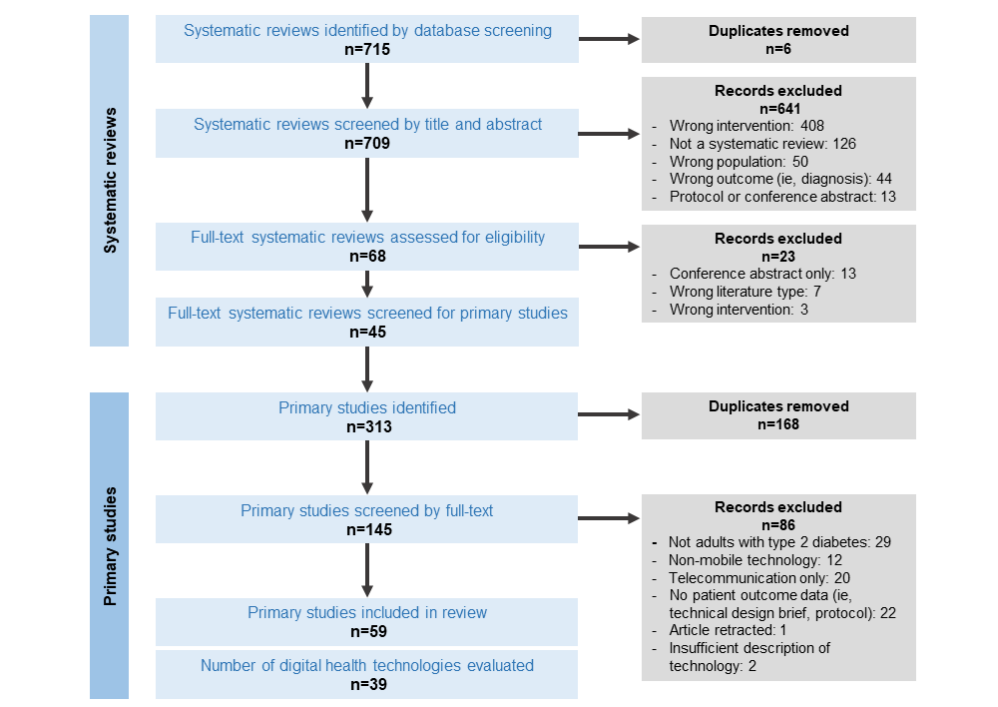
Flow diagram showing the inclusion and exclusion of systematic reviews and primary studies to yield eligible technologies.

The initial database search returned 715 citations. After removal of duplicates, 709 citations were screened by title and abstract. We identified 68 relevant systematic reviews for which we screened the full-text articles. Of these, 45 reviews were included (Figure 1).

### Screening for Primary Studies and Technologies

From these 45 reviews, we identified 145 relevant primary studies and screened their full-text articles. Of these, 61 primary studies met the inclusion criteria described above. We subsequently excluded 2 studies because there was insufficient information describing their technology to allocate a tier. The remaining 59 studies described 39 unique technologies and were included for data extraction (Figure 1).

The characteristics of the 59 included studies are presented in Multimedia Appendix 3 [19-77]. The publication year of the included studies ranged from 2007 to 2017. Of the included 59 studies, 36 (61%) were RCTs (of which 7 were identified as feasibility or pilot studies) and 23 (39%) were observational cohort studies (of which 19 were identified as feasibility or pilot studies). Qualitative data were reported alongside 6 RCTs and 13 observational cohort studies. The study nation varied, with 23 studies conducted in the United States, 6 in Norway, 4 in Korea, 3 studies each in Canada, the United Kingdom, and Saudi Arabia, 2 studies each in the Netherlands, Japan, Iran, and India, and 1 study each in Singapore, Mexico, Finland, Iraq, Bangladesh, the Democratic Republic of Congo, and China. Of the 39 technologies included for data analysis, 17 (44%) were mobile apps, 2 (5%) were personal digital assistant apps, and 20 (51%) were automated SMS.

### Assigning Technologies to an Intervention Tier

All DHTs identified and included in this review were classified as tier 3 technologies. Descriptions of the technologies and their assigned subtiers are presented in Table 1 for tier 3a and Table 2 for tier 3b.

Of the 39 technologies, 23 (59%) were assigned to tier 3a. Tier 3a describes DHTs used for preventing and managing diseases and is divided into *preventative behavior change* and *self-manage*. Of these 23 technologies, 6 were apps and 17 were SMS based. Of the tier 3a technologies, 12 were classified as *preventative behavior change* only, 3 were classified as *self-manage* only, and 8 had both 3a *preventative behavior change* and *self-manage* characteristics.

We assigned 16 (41%) of the 39 technologies to tier 3b. Tier 3b describes technologies used as tools for treatment, diagnosis, and management decisions and is divided into *treat*, *active monitoring*, *calculate*, and *diagnose*. Of these 16 technologies, 13 were apps and 3 were SMS based. Of the tier 3b technologies, 7 were *active monitoring* only, 3 were *treat* and *active monitoring*, 1 was *treat* and *calculate*, 1 was *active monitoring* and *calculate*, and 4 had all 3 of the 3b *treat*, *active monitoring*, and *calculate* characteristics.

**Table 1:** Tier 3a digital health technologies: descriptions and subtier allocation (N=23).

Digital health technology and description				Self-manage		PBC^a^
**Tier 3a app technologies**						
	Diabetes Pilot [19-22]	PDA^b^ app: patient inputs health data, displayed graphically, optionally sent to HCP^c^	✓^d^		N/A^e^	
	Few Touch App (FTA) [23-28]	Mobile app: patient inputs health data, displayed graphically. Features: personal goal setting, general diabetes information	✓		N/A	
	Unnamed (Sevick) [29]	PDA app: patient inputs diet data, feedback on nutritional composition. Features: calorie target goal set by HCP, no data access	✓		N/A	
	Monica [30]	Mobile app: patient inputs data, displayed graphically, automatic informational and/or behavioral skills feedback	✓		✓	
	iDecide [31]	Mobile app: patient inputs HbA_1c_^f^ at start. Features: education, personalized complication risk, medication review, personalized goals	✓		✓	
	Diabetes 101 [32]	Mobile app: no data input by patient. Features: 5 educational T2DM^g^ self-management videos with quiz. Automatic self-care reminders	N/A		✓	
**Tier 3a SMS technologies**						
	NICHE system [33]	SMS: patients upload BG^h^ and pedometer data onto web server: SMS summary to patient	✓		✓	
	Unnamed (Shetty) [34]	SMS: unidirectional nonpersonalized SMS (every third day), informing and reinforcing health behaviors	N/A		✓	
	Diabetech [35]	SMS: BG automatically uploaded to server: automated SMS summary, suggestions to contact HCP where relevant	✓		✓	
	Unnamed (Goodarzi) [36]	SMS: unidirectional nonpersonalized SMS (weekly) informing and reinforcing health behaviors	N/A		✓	
	Real-Time Medication Monitoring [37,38]	SMS: unidirectional SMS reminder if oral antidiabetic medication not taken (linked to electronic medication dispenser)	N/A		✓	
	Care4Life [39,40]	SMS: unidirectional nonpersonalized daily SMS, informing and reinforcing health behaviors. Two-way messaging to HCP for feedback	✓		✓	
	SMS-DMCare [41]	SMS: SMS medication reminders, unidirectional informational texts weekly about health behaviors and appointment reminders	✓		✓	
	MEssaging for Diabetes (MED) [42]	SMS: unidirectional informational SMS on medications and bidaily SMS requesting adherence response (yes or no). HCP call every 2 weeks	N/A		✓	
	TExT-MED [43,44]	SMS: unidirectional nonpersonalized bidaily SMS informing and reinforcing health behaviors	N/A		✓	
	Unnamed (Haddad) [45]	SMS: unidirectional nonpersonalized weekly SMS informing and reinforcing health behaviors	N/A		✓	
	Unnamed (Argay) [46]	SMS: unidirectional medication reminder SMS (up to 3 times daily)	N/A		✓	
	Unnamed (Bin Abbas) [47]	SMS: unidirectional nonpersonalized daily SMS informing and reinforcing health behaviors	N/A		✓	
	Unnamed (Islam) [48]	SMS: unidirectional nonpersonalized SMS every other day informing and reinforcing medication compliances	N/A		✓	
	Text to Move [77]	SMS: patient self-uploads pedometer data: 2 unidirectional text messages daily based on step count and preset goals	✓		✓	
	Unnamed (Peimani) [49]	SMS: unidirectional SMS informing and reinforcing health behaviors. Personalized to individual at start of study	N/A		✓	
	Unnamed (Fang) [50]	SMS: unidirectional nonpersonalized SMS informing health behaviors	N/A		✓	
	Dulcedigital [51]	SMS: unidirectional nonpersonalized SMS 2-3 daily reinforcing health behavior. Patient inputs BG in SMS which alerts HCP if abnormal	✓		✓	

^a^PBC: preventative behavior change.

^b^PDA: personal digital assistant.

^c^HCP: health care professional.

^d^Digital health technology falls within the subtier.

^e^N/A: not applicable.

^f^HbA_1c_: glycated hemoglobin.

^g^T2DM: type 2 diabetes mellitus.

^h^BG: blood glucose.

**Table 2:** Tier 3b digital health technologies: descriptions and subtier allocation (N=16).

Digital health technology and description				Treat		Active monitoring		Calculate
**Tier 3b app technologies**								
	BP^a^ telemanagement [52]	Mobile app: patient BP automatically uploaded. HCP^b^ accesses all data. Alert to patient and HCP if critical. Automatic BP reminders to patient	N/A^c^		✓^d^		N/A	
	WellDoc [53-58]	Mobile app: patient BG automatically uploaded, medication dose and diet self-inputted: automated personalized feedback on medication dose and behavior. HCP accesses all data	✓		✓		✓	
	t+ Diabetes [59-61]	Mobile app: patient BG automatically uploaded and insulin dose self-inputted: displayed graphically, decision aids for self-titration. HCP accesses all data and messages through the app	N/A		✓		N/A	
	Mobil Diab [62]	Mobile app: patient BG automatically uploaded, displayed graphically. HCP accesses all data and sends feedback through the app	N/A		✓		N/A	
	Health Coach App [63,64]	Mobile app: patient self-inputs health data: displayed graphically. Goal setting function. HCP accesses all data, individualized feedback, and two-way communication through the app	N/A		✓		N/A	
	Dialbetics app [65,66]	Mobile app: patient self-inputs BG data: behavioral feedback and alerts if abnormal. HCP accesses all data; abnormal readings flagged. Features: later version includes dietary feedback	✓		✓		N/A	
	SANAD [67]	Mobile app: BG^e^ automatically uploaded. Features: social networking module and CBT^f^ module. HCP accesses all data; sends feedback through app	✓		✓		N/A	
	SAED system [68]	Mobile app: BG automatically uploaded. Features: weekly educational message. HCP accesses all data; two-way communication through the app	N/A		✓		N/A	
	Diabetes Pal [69]	Mobile app: patient self-inputs BG: app suggests insulin dose (within the preset range). Features: educational information. Research staff access all data; flag to HCP	✓		N/A		✓	
	CollaboRhythm [70]	Mobile app: patient self-inputs medication and BG displayed graphically. HCP accesses all data and suggests insulin correction; two-way communication through the app	✓		✓		✓	
	PSDCS [71]	Mobile app: BG automatically uploaded, diet and exercise self-inputted—feedback and suggested insulin changes based on algorithm. Features: automated daily recommendations for calorie intake and exercise	✓		✓		N/A	
	Brew app [72]	Mobile app: patient self-inputs health data. Features: daily SMS reminders, educational information. HCP accesses summary of data and sends alerts for BG or missed appointments	N/A		✓		N/A	
	Gather Health [73]	Mobile app: patient self-inputs BG: displayed graphically. Features: daily reminders and self-care advice. HPC accesses all data; two-way communication through the app	N/A		✓		✓	
**Tier 3b SMS technologies**								
	UCDC system [74]	SMS: patient BG automatically sent to server, automated summary SMS with behavioral suggestions. Patient sends BP and exercise via SMS. Informational SMS trice daily. HCP accesses all data	N/A		✓		N/A	
	Unnamed SMS (Kim) [75]	SMS: patient BG automatically sent to server, automated SMS suggestions to adjust insulin based on an algorithm. If hypoglycemic, emergency SMS sent to patient and caregiver	✓		✓		✓	
	CDSS u-health care [76]	SMS: Patients BG automatically uploaded to server, automated daily SMS summaries, suggestions to adjust insulin based on algorithm, weekly and monthly summaries	✓		✓		✓	

^a^BP: blood pressure.

^b^HCP: health care professional.

^c^N/A: not applicable.

^d^Digital health technology falls within the subtier.

^e^BG: blood glucose.

^f^CBT: cognitive behavioral therapy.

### Assessment of Evidence According to Tier

The assessment of evidence level according to the assigned tier is presented in Table S1 [22,28-36,38,39,41-43,45-51,77] in Multimedia Appendix 4 for tier 3a technologies and in Table S2 [52,54,61,62,64,65,67-76,78] in Multimedia Appendix 4 for tier 3b technologies. Across all 39 technologies, 11 demonstrated *best practice* standards for the evidence level assigned, 3 technologies demonstrated *minimum* standards, and 25 did not report methods or findings that met *minimum* standards.

#### Tier 3a Technologies

Of the 23 tier 3a technologies, 7 met the *best practice* standards, 3 met the *minimum* evidence standards, and 13 did not report methods or findings reaching *minimum* standards. Of the 13 technologies that did not provide evidence for *minimum* standards, there were several common reasons for falling short of the *minimum* standard. First, 7 technologies did not provide statistical justification of sample size where the study design was appropriate, with this being the only reason for not meeting minimum standards in all 7 technologies. Second, 6 technologies did not provide comparative data, with this being the only reason for not meeting the minimum standards in the 2 technologies. Finally, 3 technologies did not conduct any statistical testing on the data set.

For the 3 tier 3a technologies that met the minimum evidence standards, there were 2 common reasons why these technologies did not meet the *best practice* standards. First, 2 technologies showed no improvement in condition-relevant outcomes, with this being the only reason for both technologies not meeting the best practice. Second, 1 technology’s comparator group did not represent usual care, with this being the only reason for not meeting the best practice.

#### Tier 3b Technologies

Of the 16 tier 3b technologies, 4 met *best practice* standards, none met only *minimum* evidence standards, and 12 did not report methods or findings reaching *minimum* standards. Of the 12 technologies that did not provide evidence for *minimum* standards, there were several common reasons for falling short of the *minimum* standard. First, 3 technologies used a single-arm cohort study design that lacked a comparator group and failed to meet the requirement of design being *quasi-experimental* or higher, with inappropriate study design being the only reason for not meeting minimum standards in all 3 technologies. Second, 7 technologies had no statistical justification of sample size where the study design was appropriate, with this being the only reason for 5 of these technologies. Third, there were 2 technologies that did not conduct any statistical testing on the data set. Finally, 2 technologies had a follow-up period of less than 3 months, which is the accepted minimum *clinically relevant* follow-up period for type 2 diabetes.

#### Evidence Standard by Host Country

Table 3 shows the DHTs arranged according to the income status (as defined by the World Bank [16]) of the study nation and the outcome of the DHT’s NICE evidence assessment. There were considerably more DHTs from high-income economies (n=30) than upper middle-income (n=5), lower middle-income (n=3), or low-income (n=1) economies. In addition, there was no evidence of studies from high-income nations being more or less successful in meeting NICE evidence standards than lower-income nations: only 9 out of 30 DHTs investigated in high-income economies met either *minimum* or *best practice* standards, compared with 3 out of 5 DHTs investigated in upper middle-income economies, 2 out of 3 DHTs investigated in low- and middle-income economies, and 0 out of 1 DHTs investigated in low-income economies.

**Table 3:** Digital health technologies arranged by World Bank income status of host country and the digital health technology evidence outcome (N=39).

Country		DHT^a^	NICE^b^ evidence level met
**Low-income economies**			
	Democratic Republic of Congo	Mobil Diab	No
**Lower middle-income economies**			
	Bangladesh	Unnamed (Islam)	Best practice
	India	Unnamed (Shetty)	No
	India	Gather Health	Best practice
**Upper middle-income economies**			
	China	Unnamed (Fang)	Minimum
	Iran	Unnamed (Haddad)	No
	Iran	Unnamed (Goodarzi)	Best practice
	Iraq	Unnamed (Peimani)	Best practice
	Mexico	Brew app	No
**High-income economies**			
	Canada	BP telemanagement	No
	Canada	Health Coach App	No
	Finland	Monica	No
	Hungary	Unnamed (Argay)	No
	Japan	Dialbetics app	Best practice
	Korea	CDSS-based u-health care	No
	Korea	PSDCS	No
	Korea	UCDC system	No
	Korea	Unnamed (Kim)	Best practice
	Netherlands	Real-Time Medication Monitoring	No
	Norway	Few Touch Application	Minimum
	Saudi Arabia	SANAD	No
	Saudi Arabia	SAED	No
	Saudi Arabia	Unnamed (Bin Abbas)	No
	Singapore	Diabetes Pal	No
	United Kingdom	t+Diabetes	No
	United States	Care4life	No
	United States	CollaboRhythm	No
	United States	Diabetech	No
	United States	Dulcedigital	No
	United States	Diabetes 101	No
	United States	MED	No
	United States	NICHE system	No
	United States	SMS-DMCare	No
	United States	Unnamed (Sevick)	Minimum
	United States	Diabetes Pilot	Best practice
	United States	iDecide	Best practice
	United States	TExT-MED	Best practice
	United States	Text to Move	Best practice
	United States	WellDoc	Best practice

^a^DHT: digital health technology.

^b^NICE: National Institute of Care Excellence.

## Discussion

### Principal Findings

We aimed to evaluate whether peer-reviewed literature investigating the use of mobile device DHTs for the self-management of T2DM met the required evidence level set out in the NICE Evidence Standards Framework for DHTs. The framework aims to ensure that new technologies introduced to clinical health care settings are effective and offer economic value. We identified 39 mobile device DHTs designed to support self-management of T2DM in the scientific literature; these were a mix of app-based and SMS-based technologies. We found that all technologies fell into tier 3a or tier 3b (the highest tiers) of the NICE framework, with tier 3 interventions targeting disease management and requiring the most rigorous evidence. When assessing a technology using the NICE Evidence Standards Framework, we assessed all primary studies supporting a DHT individually against the framework and selected the strongest supporting evidence for the technology reported across the primary studies.

For more than half of the technologies identified, the underpinning literature did not meet the evidence standards to demonstrate effectiveness, as recommended by the NICE framework for the technology’s tier. Of the 39 technologies identified, only 16 met *minimum* or *best evidence* standards, with 23 not meeting the minimum requirements. The most common reasons for not meeting the NICE standards included a lack of an appropriate comparator group that reflected usual care, no statistical justification of sample size, a lack of measurable improvement in condition-related outcomes, and no statistical data analysis. Given the high proportion of RCTs among the identified studies (36/59, 61%), it was surprising that such a large number did not meet the minimum evidence standards due to these reasons. We found that the evidence framework could easily be applied to a variety of study nations and that studies from a range of economic settings were able to meet evidence standards for the DHT. From the results of this study, we suggest that the application of DHT evidence standards are globally relevant.

### Using the NICE Evidence Standards Framework to Evaluate Evidence

We encountered several challenges in interpreting and using the NICE framework. First, we found that for diabetes, there was ambiguity in distinguishing technology for *healthy living* and technology for *disease management*. The same technology that targeted diet and exercise could be considered tier 2 for people without diabetes as a *healthy living app* but tier 3 for those with T2DM as a *disease management* app. There are several terms used in the NICE framework that can be ambiguous in their application and may require greater clarity, including the phrases *high quality data* and *clinically relevant follow-up period*. The framework does not include guidance as to how either of these points should be assessed.

As the NICE Evidence Framework was designed in the United Kingdom, the standards reference the UK health care setting when assessing the development and effectiveness of a technology. We found that adaptation of the NICE framework to assess a DHT in its *host country*, rather than specifically in the United Kingdom, allowed the analysis and comparison of DHTs in an international context. We also noted that the UK-specific requirement may restrict UK policy makers, commissioners, and clinicians from adopting and implementing DHTs that have been rigorously evaluated in another health care setting and do not require substantial adaptation. This could be considered overly restrictive for DHTs that target self-management and may not need integration with a health care system.

Finally, we observed a potential mismatch between the level of risk associated with an intervention and the level of evidence required according to the intervention’s associated tier. For example, Real-Time Medication Monitoring [37,38], which would be categorized under tier 3a (*preventative behavior change* due to explicit suggestions by the DHT to the patient for actions or behavior change) might be considered a low-risk technology, involving automatic SMS reminders to take medication when a patient’s pill box remains unopened. However, Health Coach App [63,64], also classified under tier 3a (*self-management* for symptoms, health or disease related data, or medication tracking over time) might be considered as having higher risk, tracking multiple health behaviors, holding sensitive data, and facilitating two-way messaging. Despite this difference in the level of risk, both technologies fall under the same tier and require the same standard of supporting evidence. The evidence framework also stipulates that any technology where there is automatic transfer of data (regardless of type) to a health care professional should be categorized as tier 3b rather than tier 3a under *active monitoring*, requiring more rigorous evidence for clinical input without any apparent additional risk. Therefore, tier levels may need to be adjusted to reflect clinical risk rather than function alone.

### Strengths and Limitations

Although this is a scoping review, we took a systematic approach to identify peer-reviewed articles, adding rigor to our methods. We included reviews of all study design types, including experimental, observational, and qualitative study designs. However, while we identified several experimental and observational studies, this approach may not have captured all developmental studies and recently published studies that are less likely to be included in systematic reviews. However, we would have expected developmental studies to be cited in subsequent experimental and observational clinical studies, and we hand-searched full-text articles for such studies. We adapted our evidence assessments where appropriate (eg, excluding requirements for BCT evidence in tier 3a).

We identified technologies that have been investigated and published in the scientific literature and did not review app catalogs or commercial publications for relevant technologies. We feel this approach was appropriate, as we did not have the resources to obtain and evaluate these sources and assess the extent to which they meet evidence standards, as described in the NICE framework. In addition, although the NICE framework was developed for DHTs used in a clinical setting, we did not differentiate between commercial and commissioned DHTs in this study. However, we encountered no challenges in applying the tier 3 evidence requirements to technologies scientifically evaluated either by clinical or commercial teams; indeed, the evidence framework could be used to design studies to evaluate the use of commercial apps within a clinical setting. Although we assessed the income status of the study nation to explore the applicability of the framework in a variety of health care settings, this did not take into account the scenario where a technology was developed in a high-income country but delivered in a low-income population [31,42-44,51,63,64]. Although beyond the scope of this review, future work could explore the effect of sociodemographic factors of the target population (such as economic status, access to health care, and technology literacy) in using the framework to evaluate the effectiveness of DHTs.

Due to potential ambiguity and subjectivity applying the NICE framework, we acknowledge that our interpretation will have affected decisions around classification and evidence evaluation and consequently the number of DHTs meeting evidence standards. We have highlighted that greater clarity of key terms in the framework would be valuable. We also acknowledge that the scope of our analysis was limited to the evidence requirements in the NICE framework, but other considerations for study quality (ie, prospective registration, retention rate) and intervention effect (ie, technology literacy, impact on behavior) are interesting and relevant in evaluating the effectiveness of DHTs.

We identified several evidence-level criteria as described by NICE that studies of DHTs commonly failed to meet. This offers a useful resource for digital health researchers and developers who may use this information in designing and reporting DHT research in the future. This might aid in the translation of research into clinical care by ensuring that the required information is measured and reported. This in turn will enable commissioners, policy makers, and clinicians to readily assess whether a technology is suitable for implementation in the UK health care setting.

### Comparison With Previous Work

Previous studies have identified a lack of evidence of an effect in apps for diabetes. Recently, Veazie et al [79] identified 15 studies evaluating 11 apps for the self-management of diabetes and found that only 5 technologies were supported by evidence showing significant clinical improvement with use. Our study supported this finding as well as identifying many more apps and several other aspects of evidence that could be improved. In addition, a previous study highlighted challenges in applying the NICE Evidence Framework tiers in classifying DHTs. Nwe et al [80] used the NICE framework to classify 76 apps from the National Health Service (NHS) app library into their relevant technology tier and assessed the classification agreement between 2 mobile health (mHealth) researchers. They found a disagreement on the classified tier in 45% (34/76) of technologies [80]. Our study complements the author’s recommendation that greater clarity in the framework may be needed to improve the consistency of its application. To our knowledge, this is the first study to assess the evidence supporting DHTs against the NICE Evidence Framework. Previous reviews evaluating DHTs in other clinical settings, such as technologies for stroke rehabilitation and virtual reality tools in pediatric care, have highlighted the need for a set of recognized standards in the field with specific mention to the NICE framework [81,82]. Therefore, it would be of interest to assess and compare the application of the NICE framework with DHTs in other health care settings in addition to chronic disease management. Given that the NICE framework is relatively new, it would be valuable to conduct similar reviews in the future to assess the potential impact of the framework on rigor and quality of studies over time.

### Conclusions

This review evaluated a defined group of mobile-delivered DHTs designed for use by people with T2DM, using the NICE Evidence Standards Framework for DHTs. Over half of the identified DHTs did not meet the minimum evidence standards required for their intervention tier, as defined by the NICE Evidence Standards Framework**.** This may pose a major barrier to the translation of mHealth interventions into the UK health care setting. However, we have highlighted the most common areas in which DHT evaluations do not meet the standards set out by NICE, and this provides an opportunity for researchers and DHT developers to address these points when designing and reporting DHTs in the future. In addition, we identified the potential scope for development of the NICE framework so that the evidence tiers correlate more closely with the associated risk of an intervention. Above all, commissioners, clinicians, and patients need to have confidence in the safety of DHTs for these to be implemented into everyday chronic disease management, and increased risk should be underpinned by the most rigorous scientific research.

## Appendix

Multimedia Appendix 1 Example of full search strategy for the Medline database.

Multimedia Appendix 2 An explanation of the classification strategy for digital health technologies using the technology tier and evidence level in the National Institute of Health and Care Excellence Framework.

Multimedia Appendix 3 Characteristics of primary studies included for data extraction.

Multimedia Appendix 4 Overall technology assessments against the National Institute for Health and Care Excellence Evidence Framework.

## Abbreviations

BCT: behavior change technique
DHT: digital health technology
mHealth: mobile health
NICE: National Institute of Care Excellence
NIHR: National Institute for Health Research
NHS: National Health Service
RCT: randomized controlled trial
T2DM: type 2 diabetes mellitus
WHO: World Health Organization
