# Supplementary material for: Application of the National Institute for Health and Care Excellence Evidence Standards Framework for Digital Health Technologies in Assessing Mobile-Delivered Technologies for the Self-Management of Type 2 Diabetes Mellitus: Scoping Review
Source: JMIR Diabetes. 2021 Feb 16;6(1):e23687. doi: 10.2196/23687 (PMC7925151; doi:10.2196/23687)
Supplement: Multimedia Appendix 1 [file diabetes_v6i1e23687_app1.pdf]

Multimedia Appendix: example of full search strategy for the Medline database

This is a Multimedia Appendix to a full manuscript published in the J Med Internet Res. For full copyright and citation information see http://dx.doi.org/10.2196/jmir.23687

# ▲	Searches
1	exp Diabetes Mellitus, Type 2/
2	((type 2 or type ii or noninsulin dependent or non-insulin dependent or mature onset or adult onset) adj3 diabet*).ti,ab.
3	*Diabetes mellitus/
4	diabet*.ti.
5	2 or 3 or 4 or 5
6	exp Telemedicine/
7	electronic mail/ or exp telephone/ or videoconferencing/
8	exp Computers, Handheld/
9	exp Internet/
10	Software/ or Mobile Applications/
11	Wearable Electronic Devices/
12	(tele-med* or tele-health* or tele-monitor* or tele-care or tele-rehab* or tele-conferen* or tele-consult* or telemed* or telehealth* or telemonitor* or telecare or telerehab* or teleconferen* or teleconsult* or mhealth or m-health or mobile health or digital health or ehealth or e-health).ti,ab.
13	((internet* or digital* or electronic or web* or online* or internet or video* or telephone* or phone* or cellphone*) adj5 (monitor* or conferen* or consult* or rehab*)).ti,ab.
14	((internet* or digital* or electronic or web* or online* or internet or video* or telephone* or phone* or cellphone*) adj intervention?).ti,ab.
15	((internet* or web*) adj based).ti,ab.
16	((remote or distan*) adj3 (monitor* or conferen* or consult* or rehab*)).ti,ab.
17	(internet* or digital* or electronic or web* or online* or internet or video* or telephone* or phone*).ti.
18	(((mobile or online or cellphone? or phone? or tablet? or digital or electronic or web*) adj2 (application? or device?)) or app or apps).ti,ab.
19	(textmessag* or text messag* or sms or cellphone* or cell phone* or mobile phone* or smartphone* or smart phone* or iphone* or i-phone* or ((tablet or handheld or hand-held) adj3 (device? or computer?)) or ipad* or i-pad*).ti,ab.
20	(wearable? adj3 (devic? or technolog* or monitor*)).ti,ab.
21	wearable.ti. or wearables.ti,ab.
22	(smart-watch* or smart-monitor* or smart-track* or smart-phone? or smart-mobile? or smart-home? or smartwatch* or smartmonitor* or smarttrack* or smartphone? or smartmobile? or smarthome?).ti,ab.
23	7 or 8 or 9 or 10 or 11 or 12 or 13 or 14 or 15 or 16 or 17 or 18 or 19 or 20 or 21 or 22 or 23
24	6 and 24
25	exp animals/ not humans.sh.
26	(comment or editorial or letter or news or review or case report).pt.
27	26 or 27
28	25 not 28
29	(systematic review or meta analysis).pt.
30	(MEDLINE or search* or meta-analysis or (systematic and (review or overview))).tw.
31	(evidence synthesis or realist synthesis or realist review).ti,ab.
32	(Qualitative and synthesis).ti,ab.
33	(meta-synthesis* or metasynthesis).ti,ab.
34	(meta-ethnograph* or metaethnograph*).ti,ab.
35	(meta-study or metastudy).ti,ab.
36	30 or 31 or 32 or 33 or 34 or 35 or 36
37	29 and 37
38	limit 38 to (english language and yr="2000 -Current")
