# Supplementary material for: Application of the National Institute for Health and Care Excellence Evidence Standards Framework for Digital Health Technologies in Assessing Mobile-Delivered Technologies for the Self-Management of Type 2 Diabetes Mellitus: Scoping Review
Source: JMIR Diabetes. 2021 Feb 16;6(1):e23687. doi: 10.2196/23687 (PMC7925151; doi:10.2196/23687)
Supplement: Multimedia Appendix 2 [file diabetes_v6i1e23687_app2.pdf]

Multimedia Appendix: An explanation of the classification strategy for digital health technologies using the technology tier and evidence level in the National Institute of Health and Care Excellence Framework

This is a Multimedia Appendix to a full manuscript published in the J Med Internet Res. For full copyright and citation information see http://dx.doi.org/10.2196/jmir.23687

Background

The National Institute for Health and Care Excellence (NICE) Evidence Standards Framework assigns digital health technologies (DHTs) different tiers (1, 2, 3a and 3b) according to their functionality [4]. Tiers are decided by factors such as measurable outcome(s) on users, independence from healthcare professionals and extent of influence over disease management, treatment and diagnosis. Each tier has a specific evidence standard needed to meet requirements, with DHTs falling into higher tiers requiring more stringent evidence to support effectiveness. This evidence requirement is cumulative, such that a DHT falling into tier 3 must also meet requirements for tier 1 and tier 2.

The NICE framework further splits tiers 1, 2, 3a and 3b into sub-classifications according to the DHT's specific functionality. With the exception of tier 3a *self-management*, sub-classifications do not affect the overall evidence requirement for a DHT but help guide which tier a technology falls within.

Assigning tiers to technology

Background

All DHTs in this study were classified as tier 3 technologies. Tier 2 technologies are described as being *unlikely to have measurable user outcomes*. As all DHTs assessed here were designed to prevent, manage or treat an aspect of disease, it was decided that this qualified them as tier 3 technologies. There was some complication here in interpreting the phrase *measurable user outcomes* in the guidelines; as we were evaluating clinical studies that assessed an effect on a population, there was an inherent *measurable user outcome* by nature of the clinical study design as opposed to any inherent factor of technology itself. This issue is touched on in the discussion.

Tier 3 technologies are split into 3a and 3b technologies, with further functional classification given below [4]:

EVIDENCE TIER	FUNCTIONAL CLASSIFICATION	DESCRIPTION	INCLUDES<br>(for example)	EXCLUDES<br>(for example)
**Tier 3a:**<br><br>DHTs for preventing and managing diseases. They may be used alongside treatment and will likely have measurable user benefits	*Preventative behaviour change*	- Designed to change user behaviour related to health issues with, for example: smoking, eating, alcohol, sexual health, sleeping & exercise.<br>- Prescribed to users by professional	- Smoking cessation DHTs and those used as part of weight loss programmes<br>- DHTs marketed as aids to good sleep habits	- DHTs that describe themselves as a treatment for a diagnosed condition<br>- Apps that provide general healthy lifestyle advice
	Self-manage	Aims to help people with a diagnosed condition to manage their health<br>- May include symptom tracking function that connects with a healthcare professional	- DHTs that allow users to record, and optionally to send data to a healthcare professional to improve management of their condition	- DHTs that describe themselves as a treatment for a diagnosed condition<br>- Apps that automatically monitor and report data to a healthcare professional or 3rd party organisation
**Tier 3b:**<br><br>DHTs with measurable user benefits, including tools used for treatment and diagnosis, as well as those influencing clinical management through active monitoring and calculation.<br>It is possible that DHTs in this tier will qualify as medical devices.	*Treat*	Provides treatment for a diagnosed condition (such as CBT for anxiety) or guides treatment decisions	- DHTs for treating mental health or other conditions<br>- Clinican-facing apps that advise on treatments in certain situations	- Apps that provides general health advice or advice with a diagnosed condition<br>- DHTs that offer general advice for clinicans such as online textbooks of digital versions of care pathways
	Active monitoring	Automatically records health information and transmits the data to a professional, carer or third-party organisation, without any input from the user.	- DHTs linked to devices such as implants, sensors worn on the body or in the home.<br>- Data are automatically transmitted through the DHT for remote monitoring.	- DHTs that allow a user to choose if and when to send recorded data to a professional, carer or third-party organisation
	Calculate	Tools that perform clinical calculations that are likely to affect clinical care decisions	- DHTs for use by clinicians, professionals or users to calculate parameters pertaining to care, such as early warning system software.	- DHTs that diagnose or provide treatment for a condition
	Diagnose	Uses data to diagnose a condition in a patient, or to guide a diagnostic decision made by a healthcare professional	- DHTs that diagnose specified clinical conditions using clinical data	- DHTs that offer general lists of signs and symptoms for healthcare conditions

Interpretation of guidelines for this study

In interpreting these guidelines, we created the following guide specific to type 2 diabetes mellitus (T2DM) mobile-delivered DHTs to use as a guide for consistency in assigning tiers:

TIER 3a	
**Preventative behaviour change**	Explicit suggestion by the DHT to the patient for actions or behaviour change
**Self-manage**	Symptom, health or disease related data, or medication tracking over time

TIER 3b	
**Treat**	Application suggests a specific change in medication (or treatment) directly to the patient
**Active monitoring**	Clinician or health care professional has unrestricted access to patient's data
**Calculate**	Performs a calculation on parameters to decide if they are outside normal
**Diagnose**	[Not relevant due to study selection criteria of a pre-existing diagnosis of T2DM]

Further interpretations of the guidelines for this study:

- Where a DHT satisfied criteria for more than one sub-classification within the same tier, both were reported in Table 1; with the exception of *preventative behaviour change* for tier 3a, this had no effect on the overall evidence requirements for a DHT.
- Where a DHT satisfied criteria for both tier 3a and 3b, only tier 3b was applied to the DHT; this was rationalised because tier 3b had a more stringent requirement in terms of evidence than tier 3a.
- Although evidence requirements are cumulative, we only assessed the topmost tier. This was a study design decision; as there was considerable overlap with lower tiers, the decision was that the extra evidence added from considering all previous tiers was small compared to only assessing the highest tier applicable.
- For DHTs where most functionalities fulfilled tier 3a requirements and only a small portion of the DHT warranted tier 3b assessment, tier 3b criteria were still adopted. This may be considered a limitation of our study methodology in being perhaps too stringent, but also provided an interesting critique on the tier guidelines. For example, for many DHTs that were data monitoring technologies principally falling under tier 3a, unlimited access to patient's data by the clinician qualified the DHT for tier 3b under *active monitoring*; thus clinical input actually incurred a greater evidence requirement than it would have otherwise, potentially counterintuitively.

Evidence assessment:

Background

According to the NICE guidelines, evidence requirements for each tier are split into *best practice* and *minimum* standards. This division is laid out in the guidelines such that DHTs with an inherent *higher risk* to users must require *best practice* rather than *minimum* evidence to meet requirements.

A risk assessment for DHT was deemed outside the scope of this case study and as such both *minimum* and *best practice* evidence requirements were shown for each DHT, regardless of risk, in order to provide a greater depth of discussion.

Interpretation of guidelines for this study

Guidance for assessing effectiveness was provided in two places in the frameweork: in the *Evidence for effectiveness standards* tables and also in *More information on the evidence for effectiveness standards* [4]: this complicated

combining guidance, and we acknowledge that interpretation of these guidelines will have shaped the overall conclusion on meeting effectiveness standards. This is an inherent challenge in applying these guidelines. The tables below for tier 3a and 3b DHTs represent our interpretation of the guidelines and were created to unite guidance and translate it into items for data extraction.

Further Interpretation of the guidelines for this study

- Where a technology was reported in more than one primary study, we analysed each primary study separately against the framework, and selected the strongest supporting evidence for the technology reported across the primary studies. The primary studies providing the strongest evidence in support of the DHT are referenced after the DHT title in Table S1 and Table S2 in Multimedia Appendix 4.
- The guidelines for *best practice* evidence in tier 3a and 3b require comparators to reflect the current care pathway in the United Kingdom. As most studies we found were performed internationally and would have failed this criterion, we decided to assess the comparator as reflecting current care pathway of the host country, with the implications of this as a requirement in the framework touched upon in the discussion.
- Tier 3a requires evidence of a published *behavioural change technique* (BCT) in the development or use of a technology that encourages behavioural change. For the purposes of our case study, we excluded the evidence requirement for a BCT in our overall decision of whether a technology met evidence requirements. This was to take account of where development studies were not captured in our search strategy, and in recognition that this evidence requirement was dependent on our interpretation of *behaviour change*. This is touched upon in the discussion.

<table>
<tr><th colspan="3">TIER 3a: Evidence for Effectiveness Standards</th></tr>
<tr><th colspan="3">ALL 3a TECHNOLOGIES</th></tr>
<tr><th></th><th>Minimum evidence requirement</th><th>Best Practice evidence requirements</th></tr>
<tr><td>Description in the Evidence for effectiveness Table [4]</td><td>High quality observational or quasi experimental studies demonstrating relevant outcomes. These studies should present comparative data. Comparisons could include: relevant outcomes in a control group, use of historical controls, routinely collected data. Relevant outcomes may include: behaviour or condition related user outcomes such as a reduction in smoking or improvement in condition management, evidence of positive behaviour change, user satisfaction</td><td>High quality quasi experimental or experimental studies which incorporate a comparison group, showing improvements in relevant outcomes, such as: patient reported outcomes (preferably using validated tools including symptom severity or quality of life, other clinical measures of disease severity or disability, healthy behaviours, physiological measures, user satisfaction and engagement, health and social care resource use such as admissions or appointments. The comparator should be a care option that is reflective of the current care pathway, such as a commonly used active intervention.</td></tr>
<tr><td>Description in the More information section [4]</td><td>A high quality observational or quasi-experimental study would observe and clearly describe the effect of the DHT on a group of representative users, and allow some comparison with outcomes without the intervention. The study would include statistical considerations such as sample size and statistical testing, report outcomes (ideally valid and reliable outcome measures) that are relevant to the condition, and be clear on reporting the outcomes of every person in the trial.</td><td>A high quality quasi-experimental or experimental study would compare the effect of the DHT on a group of users with 1 or more groups having a different (or no) treatment. The study would report the difference between the groups. It would include statistical considerations such as sample size and statistical testing, report outcomes that are relevant to the condition, and be clear on reporting the outcomes of every person in the group testing the DHT. Ideally, the comparator group would be people having current standard care, but it could also be a before-and-after study (measuring people’s symptoms over a period of time before they use the DHT then comparing this with while they are using the DHT).</td></tr>
<tr><td>Items for data extraction</td><td>Study design:
Study design observational or higher

Comparator:
Comparative data present (control or historical control)

Outcomes:
Outcome relevant to condition measured

Relevant outcomes may include:
Behavioural or condition-related user outcomes (ie improvement in condition management)
Evidence of positive behaviour change</td><td>Study design:
Study design quasi-experimental or higher

Comparator:
Comparator reflects current standard care

Outcomes:
Improvement in condition relevant outcome

Relevant outcomes may include:
Patient reported outcomes using validated tools (ie symptom severity or quality of life)
Clinical measures of disease severity or disability</td></tr>
</table>

	• User satisfaction	• Healthy behaviours<br>• Physiological measures<br>• User satisfaction and engagement<br>• Health and social care resource use (ie number of admissions or appointments)
	**Data quality considerations - minimum and best practice:**<br>Statistically justified sample size<br>Statistical calculation performed on data<br>Clearly reports follow-up and dropout (as proxy for outcome of every person)	
**TECHNOLOGIES USING A BEHAVIOUR CHANGE TECHNIQUE (i.e. tier 3a 'Preventative Behaviour Change)**		
Description in 'Evidence for effectiveness' Table [4]	Be able to show that the techniques used in the DHT are: consistent with recognised behaviour change theory and recommended practice (aligned to guidance from NICE or relevant professional organisations); appropriate for the target population.	Published qualitative or quantitative evidence showing that the techniques used in the DHT are: based on published and recognised effective behaviour change techniques; aligned with recommended practice; appropriate for the target population.
Description in 'More information' section [4]	DHTs that aim to change the behaviour of the users should be consistent with accepted and effective behaviour change techniques. The DHT owner should be able to describe which behaviour change techniques are used and provide references to these.	**Not applicable**
**Items for data extraction**	**Appropriate Behaviour Change Techniques (BCT) - minimum and best practice:**<br>BCT relevant<br>Describes which techniques are used in relation to the app's design or functionality<br>References a published BCT or recommended practice	

<table>
<tr><th colspan="3">TIER 3b: Evidence for Effectiveness Standards</th></tr>
<tr><th></th><th>Minimum evidence requirement</th><th>Best Practice evidence requirements</th></tr>
<tr><td>Description in the Evidence for effectiveness Table [4]</td><td>High quality experimental or quasi-experimental studies showing improvements in relevant outcomes, such as: diagnostic accuracy; patient-reported outcomes including symptom severity or quality of life; other clinical measures of disease severity or disability; healthy behaviours physiological measures; user satisfaction and engagement. Generic outcome measures may also be useful when reported alongside condition-specific outcomes. The comparator should be a care option that is reflective of the current care pathway, such as a commonly used active intervention.</td><td>High quality randomised controlled study or studies done in a setting relevant to the UK health and social care system, comparing the DHT with a relevant comparator and demonstrating consistent benefit including in clinical outcomes in the target population, using validated condition-specific outcome measures. Alternatively, a well-conducted meta-analysis of randomised controlled studies if there are enough available studies on the DHT.</td></tr>
<tr><td>Description in the More information section [4]</td><td>A high quality quasi-experimental or experimental study would compare the effect of the DHT on a group of users with 1 or more groups having a different (or no) intervention. The study would report the difference between the 2 groups. The study would include statistical considerations such as sample size and statistical testing, report outcomes that are relevant to the condition, and be clear on reporting the outcomes of every person in the group testing the DHT. Ideally, the comparator group would be people having current standard care, but could also be a before-and-after study (measuring people’s symptoms over a period of time before they use the DHT then comparing this with while they are using the DHT). The outcome measures reported should reflect best practice for reporting improvements in the specific condition, using validated outcome measures such as those in the COMET core outcome set.</td><td>A high quality randomised controlled trial or trials would take a group of people with the condition and randomly assign people to use the DHT or a comparator. The people in both groups would be followed-up over a relevant period of time to compare the outcomes of the groups. The study would report the difference between the 2 groups. The study would include statistical considerations such as sample size and statistical testing, report outcomes that are relevant to the condition, and be clear on reporting the outcomes of every person in the group testing the DHT. The improvements measured should be clinically relevant. Ideally, the comparator group would be people having current standard care, but comparative outcomes could also be collected in a before-and-after study (measuring people’s symptoms over a period of time before they use the DHT then comparing this with while they are using the DHT).The outcome measures reported should reflect best practice for reporting improvements in the specific condition, using validated outcome measures such as those in the COMET core outcome set.</td></tr>
<tr><td rowspan="2">Items for data extraction</td><td>Study design:
Study design quasi-experimental or higher

Outcomes:
Outcome clinically relevant to condition

We defined clinically relevant outcomes using SCORE-IT [78] as an accepted COMET clinical outcome set for T2DM</td><td>Study design:
RCT
Relevant follow-up: >3 months*

*>3 months is a widely accepted measure of clinically relevant follow-up period in diabetic studies</td></tr>
<tr><td colspan="2">Data quality considerations - minimum and best practice:
Comparator group present (can be before and after study)
Comparator reflects current standard care
Improvement in a clinical outcome with use of the technology
Justified sample size
Statistical testing on data set
Clearly reports follow-up and dropout</td></tr>
</table>
