# Supplementary material for: Application of the National Institute for Health and Care Excellence Evidence Standards Framework for Digital Health Technologies in Assessing Mobile-Delivered Technologies for the Self-Management of Type 2 Diabetes Mellitus: Scoping Review
Source: JMIR Diabetes. 2021 Feb 16;6(1):e23687. doi: 10.2196/23687 (PMC7925151; doi:10.2196/23687)
Supplement: Multimedia Appendix 3 [file diabetes_v6i1e23687_app3.pdf]

Multimedia Appendix: Characteristics of primary studies included for data extraction

This is a Multimedia Appendix to a full manuscript published in the J Med Internet Res. For full copyright and citation information see http://dx.doi.org/10.2196/jmir.23687

Technology	Author & Year	Country	Study Design	Size[a]	Follow-up	Population	Comparator(s)	Primary outcome measure(s)
**App technologies**								
Diabetes Pilot	Forjuoh (2007)[19]	USA[b]	Cohort: 1 arm (feasibility)	43	6m[c]	General adult	None	Implementation challenges , clinical marker (HbA1C)[d]
	Forjuoh (2008)[20]	USA	Cohort: 1 arm	43	6m	General adult	None	Clinical marker (HbA1C)
	Vuong (2012)[21]	USA	RCT[e]: 4 arms	376	Unclear	Ethnically diverse	Usual care	Qual.[f] user feedback (survey)
	Forjuoh (2014)[22]	USA	RCT: 4 arms	376	24m	Ethnically diverse	Usual care	Clinical marker (HbA1C)
Few Touch Application	Arsand (2010)[23]	Norway	Cohort: 1 arm (feasibility)	12	6m	General adult	None	Qual. user feedback (interview, survey)
	Nes (2012)[24]	Norway	Cohort:1 arm (feasibility)	15	3m	General adult	None	Clinical marker (HbA1c, BMI[g]); semi-quant.[h] emotional distress (PAID) and QoL[i] (ADDQoL10)
	Tatara (2013)[25]	Norway	Cohort:1 arm	12	1yr[j]	General adult	None	Qual. user feedback (interview, survey)
	Chomutare (2013)[26]	Norway	Cohort: 1 arm (feasibility)	7	12wk[k]	General adult	None	Semi-quant. usability score (System Usability Score (SUS))
	Torbjornsen (2014)[27]	Norway	RCT: 3 arms	151	1yr	General adult	Usual care	Clinical marker (Hb1Ac)
	Holmen (2014)[28]	Norway	RCT: 3 arm	151	1yr	General adult	Usual care	Clinical marker (HbA1c)
Unamed PDA app	Sevick (2012)[29]	USA	RCT: 2 arms	296	6m	General adult	Usual care + education	Clinical markers (HbA1c + others)
Monica	Orsama (2013)[30]	Finland	RCT: 2 arms	48	10m	General adult	Usual care + education	Clinical marker (HbA1c, BP[l])
iDecide	Heisler (2014)[31]	USA	RCT: 2 arms	188	3m	Low income Latino & African American	Usual care + education	Quant.[m] knowledge test & semi-quant user-satisfaction survey

Diabetes 101	Wood (2015)[32]	USA	Cohort: 1 arm (pilot)	7	2wk	Rural population	None	Semi-quant. self-care, self-efficacy & knowledge score (survey)
BP tele-management	Logan (2007)[52]	Canada	Cohort: 1 arm (pilot)	33	4m	High BP	None	Clinical marker (BP); qual. user feedback (interview)
WellDoc	Quinn (2008)[53]	USA	RCT: 2 arms	30	3m	General adult	Usual care	Clinical marker (HbA1C); semi-quant. self-care score (SDSCA) & user satisfaction
	Quinn (2011)[54]	USA	RCT: 4 arms	163	1yr	General adult	Usual care	Clinical marker (HbA1c)
	Katz (2012)[55]	USA	Cohort: 1 arm (pilot)	32	52wk	African American	None	Usability (data entries), no.[n] hospitalisations, clinical marker (HbA1C + others), qual. user satisfaction
	Quinn (2014)[56]	USA	RCT: 2 arms	112	1yr	Medication users	Usual care	Medication prescribed (medication(s), dose, freq.[o])
	Quinn (2015)[57]	USA	Cohort: 1 arm (pilot)	7	1m	Over 65 years	None	Semi quant. self-efficacy score (Stanford Survey)
	Quinn (2016)[58]	USA	RCT: 2 arms	118	1yr	<55yrs vs >55yrs	Usual care	Clinical marker (HbA1c)
t+ Diabetes	Turner (2009)[59]	UK[p]	Cohort: 1 arm (feasibility)	23	>3m	Poor BG[q] control	None	Research challenges, qual. user feedback (survey)
	Larsen (2010)[60]	UK	Cohort: 1 arm (feasibility)	23	6m	Poor BG control	None	Clinical marker (HbA1c)
	Nagrebetsky (2013)[61]	UK	RCT: 2 arms (feasibility)	14	1yr	General adult	Usual care + phone calls	Qual. user feedback (survey); clinical marker (HbA1C)
Mobil Diab	Takenga (2014)[62]	DRC	RCT: 2 arms (feasibility)	40	2m	General adult	Usual care	Semi-quant. usabililty & acceptance (survey); clinical marker (HbA1C, mean BG)
Health Coach App	Wayne (2014)[63]	Canada	Cohort: 1 arm (pilot)	21	6m	Low-income urban	None	Implementation challenges, clinical marker (HbA1C)
	Wayne (2015)[64]	Canada	RCT: 2 arms	131	6m	Low-income urban	Usual care + health coach	Clinical marker (HbA1c)
Dialbetics app	Waki (2014)[65]	Japan	RCT: 2 arms	54	3m	General adult	Usual care	Clinical marker (HbA1C), semi quant. user feedback
	Waki (2015)[66]	Japan	Cohort: 1 arm (pilot)	5	1wk	General adult	None	Qual. user feedback (interview)

SANAD	Alanzi (2016)[67]	Saudi Arabia	Cohort: 1 arm (feasibility)	33	Unclear	General adult	None	Semi quant. user satisfaction survey (QUIS)
SAED system	Alotaibi (2016)[68]	Saudi Arabia	RCT: 2 arms (pilot)	20	6m	General adult	Usual care	Clinical marker (HbA1C), quant. knowledge test
Diabetes Pal	Bee (2016)[69]	Singapore	RCT: 2 arm (pilot)	66	24wk	Poor BG control	Usual care	Implementation challenges, clinical marker (BG)
CollaboRhythm	Hsu (2016)[70]	USA	RCT: 2 arm (feasibility)	40	12wk	Poor BG control	Usual care	Clinical marker (HbA1C), semi quant. user satisfaction (DTSQ), qualitative user feedback (interview)
PSDCS	Kim (2016)[71]	Korea	Cohort 1 arm (pilot)	29	12wk	General adult	None	Clinical marker (HbA1c) semi quant. self-care score (SDCSA)
Brew app	Anzaldo-Campos (2016)[72]	Mexico	RCT: 3 arms	301	10m	General adult	Usual care	Clinical marker (HbA1c)
Gather Health	Kleinman (2016)[73]	India	RCT: 2 arms	91	6m	General adult	Usual care	Clinical marker (HbA1c)
**SMS technologies**								
NICHE system	Faridi (2008)[33]	USA	RCT: 2 arms (pilot)	30	1yr	General adult	Usual care	Implementation challenges, semi quant. self-care score (lDSES), clinical marker (HbA1C)
Unamed SMS	Shetty (2011)[34]	India	RCT: 2 arms (pilot)	215	3m	General adult	Usual care	Semi-quant. self care activity adherence (survey), clinical marker (HbA1C),
Diabetech (GlucoMON)	Roblin (2011)[35]	USA	Cohort: 1 arm (pilot)	15	3m	Poor BG control	None	Implementation challenges, qualitative user feedback (interview)
Unamed SMS	Goodarzi (2012)[36]	Iran	RCT: 2 arms	100	6m	General adult	Usual care	Clinical markers (HbA1C + others) quant. knowledge score, semi-qual. self-efficacy (survey)
Real Time Medication Monitoring	Vervloet (2012)[37]	Netherlands	RCT: 2 arms	104	6m	Poor compliance	Non-digital pill box	Medication adherence (proportion of missed doses)
	Vervloet (2014)[38]	Netherlands	RCT: 3 arms	161	2yr	Poor compliance	Usual care	Medication adherence (prescription refill rate)
Care4Life	Georgsson (2015)[40]	USA	Cohort: 1 arm (feasibility)	10	N/A (CS)[r]	General adult	None	Qual. user feedback (interview + survey), study challenges
	Capozza (2015)[39]	USA	RCT: 2 arms	93	6m	General adult	Usual care	Clinical marker (HbA1C)

SMS-DMCare	Nundy (2012)[41]	USA	Cohort: 1 arm (pilot)	18	4wk	African-American	None	Qual. user feedback (interview)
Messaging for Diabetes	Osborn (2013)[42]	USA	Cohort: 1 arm (pilot)	20	6m	Low-income urban	None	Implementation challenges, qual. user feedback (interview)
TExT-MED	Arora (2014)[43]	USA	RCT: 2 arms	128	6m	Low-income urban	Usual care	Clinical marker (HbA1c)
	Burner (2014)[44]	USA	Cohort: 1 arm	24	6m	Low-income urban	None	Qual. user feedback (interview)
Unnamed SMS	Haddad (2014)[45]	Iraq	Cohort: 1 arm (feasibility)	50	29wk	General adult	None	Clinical marker (HbA1c)
Unnamed SMS	Argay (2015)[46]	Hungary	RCT: 2 arms	131	1yr	General adult	Usual care	Clinical marker (HbA1c)
Unnamed SMS	Bin Abbas (2015)[47]	Saudi Arabia	Cohort: 1 arm	100	4m	General adult	None	Clinical marker (HbA1c + others)
Unnamed SMS	Islam (2015)[48]	Bangladesh	RCT: 2 arms	236	6m	General adult	Usual care	Clinical marker (HbA1c)
Text to Move	Agboola (2016)[77]	USA	RCT: 2 arms	126	6m	General adult	Usual care + pedometers	Step count
Unnamed SMS	Peimani (2016)[49]	Iran	RCT: 3 arms	150	12wk	General adult	Usual care	Clinical marker (HbA1c)
Unamed SMS	Fang (2017)[50]	China	RCT: 2 arms	129	12m	General adult	Monthly phone call	Clinical marker (HbA1c + metabolic)
Dulcedigital	Fortmann (2017)[51]	USA	RCT: 2 arms	126	6m	Low-income Hispanic	Usual care	Clinical marker (HbA1c)
UCDC system	Yoo (2009)[74]	Korea	RCT: 2 arms	123	3m	General adult	Usual care	Clinical marker (HbA1c + BP)
Unamed SMS	Kim (2010)[75]	Korea	RCT: 2 arms	92	12wk	Glargine users	Usual care	Clinical marker (HbA1c)
CDSS-based u-healthcare	Lim (2011)[76]	Korea	RCT: 3 arms	154	6m	General adult	Usual care	Clinical marker (HbA1c)

[a] *Size* is number of participants recruited to study before dropout

[b] USA: United States of America

[c] m: months

[d] HbA1C: haemoglobin A1c protein

[e]RCT: randomised controlled trial

[f]qual: qualitative

[g]BMI: Body Mass Index

[h]semi quant: semi quantitive

[i] QoL: Quality of Life

[j] wk: weeks

[k] yr: years

[l]BP: blood pressure

[m]quant: quantitative

[n]no.: number

[o]freq.: frequency

[p]UK: United Kingdom

[q]BG: blood glucose

[r]CS: cross-section
