# Supplementary material for: Application of the National Institute for Health and Care Excellence Evidence Standards Framework for Digital Health Technologies in Assessing Mobile-Delivered Technologies for the Self-Management of Type 2 Diabetes Mellitus: Scoping Review
Source: JMIR Diabetes. 2021 Feb 16;6(1):e23687. doi: 10.2196/23687 (PMC7925151; doi:10.2196/23687)
Supplement: Multimedia Appendix 4 [file diabetes_v6i1e23687_app4.pdf]

Multimedia Appendix: Overall technology assessments against the National Institute for Health and Care Excellence Evidence Framework

This is a Multimedia Appendix to a full manuscript published in the J Med Internet Res. For full copyright and citation information see http://dx.doi.org/10.2196/jmir.23687

Table S1. Tier 3a digital health technologies: overall technology assessment against the National Institute for Health and Care Excellence Evidence Framework (N = 23)

Digital health technology	Minimum			Best practice			Data quality considerations [d]				Reaches NICE [f] Evidence Criteria
	Study design observational or higher	Outcome measured relevant to condition [a]	Comparative data present [b]	Design quasi experimental or higher	Improvement in clinical outcome with DHT [c]	Comparator reflects standard care	Statistically justifies sample size [d]	Statistical testing on dataset	Clearly reports follow up & dropout	Retention in absolute figures: n(%)[e]	
**Tier 3a app technologies**											
Diabetes Pilot [22]					Non- significant					325 (86%)	Best practice
Few Touch App. [28]										120 (79%)	Minimum
Unnamed (Sevick) [29]										246 (83%)	Minimum
Monica [30]					Significant					48 (86%)	No
iDecide [31]					Significant					176 (94%)	Best practice
Diabetes 101 [32]					Qualitative	No comparator	Cohort			7 (64%)	No
**Tier 3a SMS technologies**											
NICHE [33]										30 (100%)	No
Unamed (Shetty) [34]										144 (67%)	No
Diabetech [35]						No comparator	Cohort			15 (100%	No
Unnamed(Goodarzi) [36]					Significant					81 (81%)	Best practice
Real Time Medication[38]					Significant					126 (78%)	No
Care4Life [39]										93 (60%)	No
SMS-DMCare [41]					Qualitative	No comparator	Cohort			18 (95%)	No
Messaging for Diabetes [42]						No comparator	Cohort			Unclear	No
TexT-MED [43]					Non- significant					92 (72%)	Best practice
Unnamed (Haddad) [45]					Significant	No comparator	Cohort			42 (84%)	No
Unnamed (Argay) [46]										122 (93%)	No

Unnamed (Bin Abbas)[47]					Signficant	No comparator	Cohort			Unclear	No
Unnamed (Islam) [48]					Signficant					200 (85%)	Best practice
Text to Move [77]					Non- significant					95 (75%)	Best practice
Unnamed (Peimani) [49]					Signficant					150 (100%)	Best practice
Unnamed (Fang) [50]					Signficant					109 (84%)	Minimum
Dulcedigital [51]					Signficant					113 (90%	No

green: yes
red: no
blue: not applicable

[a] Relevant outcome may include: condition-related outcome, positive behaviour change, user satisfaction

[b] Comparative data may include: relevant outcomes in control group, use of historical controls, routinely collected data

[c] Relevant outcome may include: patient-reported (symptoms or quality of life), clinical measures, physiological measures, healthy behaviours, satisfaction & engagement, health resources (admission, appointment); the framework describes a need to demonstrate *improvement*, but not a need for this to be statistically significant. Here *Significant* represents studies that claimed a quantitative improvement with a DHT where reported p values were <0.05, *Non-significant* represents studies that claimed a positive trend to quantitative improvement but with p>0.05, and *Qualitative* represents studies that claimed an improvement with a DHT in terms of a qualitative metric

[d] Needed for both *minimum* & *best practice*: guidelines define *high quality data* as studies having justified sample size (ie, power calculation, other statistical means) and reported patient outcomes (ie, flowchart, dropout) ; in cohort studies, sample size was qualitatively rather than statistically justified due to the absence of a control group

[e] Retention in absolute figures describes the number of participants that were followed through to the final data collection at the end of the study period; the proportion is given in brackets

[f] NICE: National Institute for Health and Care Excellence

Table S2. Tier 3b digital health technologies: overall technology assessment against National Institute for Health & Care Excellence Evidence Framework (N = 16)

Digital health technology	Minimum: Design quasi-experimental or higher	Minimum: Outcome measured clinically relevant [a]	Best practice: RCT[b] study design	Best practice: Follow-up is a clinically relevant time period [c]	Data quality considerations [d]: Comparator group reflects standard care [e]	Data quality considerations [d]: Improvement in clinical outcome with DHT [f]	Data quality considerations [d]: Statistically justifies sample size [d]	Data quality considerations [d]: Statistical testing on dataset	Data quality considerations [d]: Clearly reports follow up & dropout	Data quality considerations [d]: Retention in absolute figures: n(%) [g]	Reaches NICE [h] Evidence Criteria
**Tier 3b app technologies**											
BP tele-management [52]					No control	Significant	Cohort			31 (94%)	No
WellDoc [54]						Significant				163 (77%)	Best practice
t+ Diabetes[61]						Non-significant				13 (93%)	No
Mobil Diab [62]						Non-significant				40 (100%)	No
Health Coach App [64]						Significant				98 (74%)	No
Dialbetics app [65]						Significant				49 (91%)	Best practice
SANAD [67]					No control	Qualitative	Cohort			33 (100%)	No
SAED system [68]						Significant				20 (100%)	No
Diabetes Pal [69]						Non-significant				Unclear	No
CollaboRhythm [70]						Significant				35 (88%)	No
PSDCS [71]					No control	Significant	Cohort			29 (97%)	No
Brew app [72]										264 (88%)	No
Gather Health [73]						Significant				80 (88%)	Best practice
**Tier 3b SMS technologies**											
UCDC system [74]						Significant				111 (90%)	No
Unnamed SMS (Kim) [75]						Significant				92 (92%)	Best practice
CDSS u-healthcare [76]						Significant				144 (94%)	No

green: yes
red: no
blue: not applicable

[a] Relevant outcomes belong to an accepted COMET core outcome set (we defined by SCORE-IT for type 2 diabetes mellitus (T2DM))[78]

[b] RCT: randomised controlled trial

[c] ≥3 months considered *clinically relevant* for T2DM

[d] Needed for both *minimum* & *best practice*: guidelines define *high quality data* as studies having justified sample size (ie, power calculation, other statistical means) and reported patient outcomes (ie, flowchart, dropout); in cohort studies, sample size was qualitatively rather than statistically justified due to the absence of a control group

[e] Comparative data may include: relevant outcomes in control group, use of historical controls, before-and-after study

[f] The framework describes a need to demonstrate *consistent benefit*, but not a need for improvement to be statistically significant. Here, *Significant* represents studies that claimed a quantitative improvement with a DHT where reported p values were <0.05, *Non-significant* represents studies that claimed a positive trend to quantitative improvement but with $p>0.05$, and *Qualitative* represents studies that claimed an improvement with a DHT in terms of a qualitative metric

[g] Retention in absolute figures describes the number of participants that were followed through to the final data collection at the end of the study period; the proportion is given in brackets

[h] NICE: National Institute for Health and Care Excellence
